# School zone road safety assessment using the iRAP Star Rating for Schools (SR4S) methodology in Khorasan Razavi Province 

**Published:** 2019-08

**Authors:** Ali Zayerzadeha, Mohsen Fallah Zavareh

**Affiliations:** ^*a*^Road Safety Pioneers NGO Director, Road Safety Expert, Khorasan Razavi RMTO, Mashhad, Iran.; ^*b*^Head of research office at Road Safety Pioneers NGO.

## Abstract

**Background:**

Abstract Students who walk to school often have to deal with traffic, weather conditions and other distractions along the way. Every hour, nearly 150 children between ages 0 and 19 are treated in emergency departments for injuries sustained in motor vehicle crashes. More children ages 5 to 19 die from crash-related injuries than from any other type of injury. In this research road safety situation around 7 primary schools along Khorasan Razavi province rural roads were assessed using the Star Rating for Schools (SR4S) methodology. Safety improvement treatments were done in 3 schools and reassessment of schools showed meaningful safety enhancement both in allocated score and star rating. Background Road crashes have been shown to be the leading cause of death of children and young adults aged 5–29 (WHO, 2018), indicating that compared to other causes of unintentional injuries, road injuries account for more years of potential life lost (YPLL) (Gardner & Sanborn, 1990 pp. 322) for the children. Estimates show that everyday more than 500 children lose their lives due to road crashes (WHO, 2015). Star Rating for Schools (SR4S) has been claimed to be the first ever systematic and evidence-based tool developed by iRAP (International Road Assessment Programme).

**Methods:**

The method is mainly based on engineering assessment of the school area at high risk locations. However, it is challenging to detect high risk locations in the lack of road crashes or in the lack of adequate accident data. For school assessment, different data for 7 schools such as traffic volume, number of students, operational speed, road width, crossing facilities, etc were gathered and using the iRAP SR4S application safety scores calculated.

**Results:**

In selected method lower score equals safer school zone. 3 schools with highest safety assessment scores were selected for treatments implementation by local road authority. Reassessment of school zones showed significant improvement in safety scores. The safety score for Shohada, Imam Ali and Azmoodeh School were 243,192 and 65 respectively that decreased to 37, 11 and 10 after treatment. In SR4S it means the start rating for these 1-star schools has been improved to 3,4 and 2-star respectively.

**Conclusions:**

Higher star for a school zone, however, means safer provided infrastructure and facilities for school students but the behavior of road users is the factor must be considered as well and education and enforcement still have their key roles for saving more lives on the road.

**Keywords:**

Student, School zone, Safety assessment, Star rating, References gardner

